# Biomechanical analysis of canine medial patellar luxation with femoral varus deformity using a computer model

**DOI:** 10.1186/s12917-020-02644-5

**Published:** 2020-12-03

**Authors:** Jiyun Lee, Heedong Sim, Jaemin Jeong, Sun-Young Kim, Seokjo Yang, SeongMok Jeong, HaeBeom Lee

**Affiliations:** 1 Department of Veterinary Surgery, College of Veterinary Medicine, Chungnam National University, 34134 Daejeon, Republic of Korea; 2 Department of Mechatronics Engineering, College of Engineering, Chungnam National University, 34134 Daejeon, Republic of Korea; 3 Department of Veterinary Clinical Sciences, College of Veterinary Medicine, Purdue University, 47906 West Lafayette, IN USA

**Keywords:** Finite element method, Medial patellar luxation, Femoral Varus deformity, Dog

## Abstract

**Background:**

Femoral varus deformities complicating the realignment of the quadriceps muscles are frequently associated with medial patellar luxation (MPL) in dogs. Therefore, distal femoral osteotomy (DFO) is recommended in dogs affected with severe MPL and a distal femoral varus deformity. The presence of an anatomic lateral distal femoral angle (aLDFA) of ≥ 102° has been anecdotally recommended as an indication for performing corrective DFO in large-breed dogs. However, the effect of a femoral varus deformity on MPL has not been scientifically evaluated. We aimed to evaluate the influence of a femoral varus deformity on MPL using a finite element method based computer model.

Three-dimensionally reconstructed computed tomographic images of a normal femur from a Beagle dog were deformed using meshing software to create distal varus deformities. A total of thirteen aLDFAs, including 95°, 98° and 100°–110°, were simulated. The patellar positions and reaction force between the patella and trochlear grooves were calculated for all finite element models under constant rectus femoris muscle activation.

**Results:**

The patella was displaced medially from the trochlear groove at an aLDFA of ≥103°. With an aLDFA of 103° to 110°, the reaction force was equal to zero and then decreased to negative values during the simulation, while other models with aLDFAs of 95°, 98°, and 100°-102° had positive reaction force values. The patella began to luxate at 24.90 seconds (sec) with an aLDFA of 103°, 19.80 sec with an aLDFA of 104°, 21.40 sec with an aLDFA of 105°, 20.10 sec with an aLDFA of 106°, 18.60 sec with an aLDFA of 107°, 15.30 sec with an aLDFA of 108°, 16.60 sec with an aLDFA of 109°, and 11.90 sec with an aLDFA of 110°.

**Conclusion:**

Severe distal femoral varus with an aLDFA of ≥103° caused MPL when other anatomical factors were controlled. Thissimplified computer model provides complementary information to anecdotal cutoffs for DFO, hence it should be applied to clinical patients with caution.

## Background

Medial patellar luxation (MPL) is a common orthopedic disease of the canine stifle [1]. MPL is considered a developmental disorder because it mostly occurs after birth or early in life without trauma, although the underlying etiopathogenesis remains unclear [2, 3]. It has been suggested that coxa vara and a small anteversion angle are the underlying causes of MPL, as they lead to the medial displacement of the quadriceps femoris muscle, which results in skeletal deformities such as distal femoral varus, femoral torsion, a shallow trochlear groove, the medial displacement of the tibial tuberosity, and internal rotation of the tibia. These anatomical abnormalities of the pelvic limb have been reported as important risk factors for the malalignment of the quadriceps mechanism [1–4].

Effective surgical correction of MPL involves realignment of the quadriceps mechanism by which the quadriceps muscles contract and transfer forces to the tibia to extend the stifle joint. Thereby the patella articulates via the trochlear groove and the patellofemoral joint is stabilized [3]. The surgical procedures generally include a combination of soft tissue and bone reconstruction, such as deepening of the femoral trochlear groove and tibial tuberosity transposition [1, 5]. The overall recurrence rate of grade 2 and 3 MPL in dogs after surgical correction is between 6 and 11%, while for grade 4 MPL, the recurrence rate is between 14 and 36% [5–7]. It has been reported that grade 4 MPL in dogs is frequently associated with severe distal femoral varus deformities, thereby complicating the realignment of the quadriceps mechanism [8–10].

Distal femoral osteotomy (DFO) is a surgical technique performed to realign the quadriceps mechanism by correcting the femoral varus deformity in MPL cases [9, 11]. Recent studies have shown good prognoses and low recurrence rates in dogs with severe MPL treated by DFO compared to previous studies in which femoral varus deformities were neither measured nor treated [5–7, 9, 11–13]. Several studies have suggested considering corrective osteotomy in small-breed dogs with significant distal femoral varus deformities [5, 8, 10]. The presence of an anatomic lateral distal femoral angle (aLDFA) of ≥ 102° or a femoral varus angle (FVA) of ≥ 12° has been anecdotally recommended as an indication for performing corrective DFO in large-breed dogs [11, 13]. However, the effect of femoral varus deformities on MPL has not been scientifically evaluated. Moreover, the cutoff of aLDFA for DFO remains controversial despite the presence of anecdotal recommendations [9, 14].

The finite element method (FEM) is a computer-based modeling and simulation technique that has been used for the evaluation of procedures and conditions in orthopedics, such as different types of implants, surgical techniques, and pathologies [15]. In FEM, a complex geometric shape is modeled as a mesh of simpler structures (finite elements), each having appropriate biological material properties, such as the appropriate density and modulus of elasticity of muscle and bone [16]. Therefore, FEM is suitable for parametric analyses in which the effect of specific parameters are investigated in a controlled manner [16]. FEM has been widely used in human medicine as an alternative to cadaveric and in vivo biomechanical experiments [17–19]. Recently, FEM has also been increasingly frequently used in veterinary medicine [20, 21].

The objective of the present study was to evaluate the influence of femoral varus deformities on patellar alignment using a computer model. We hypothesized that an increase in aLDFA would increase the tendency of the MPL in the computer models.

## Results

### Luxation of the patella

**Fig. 1:**
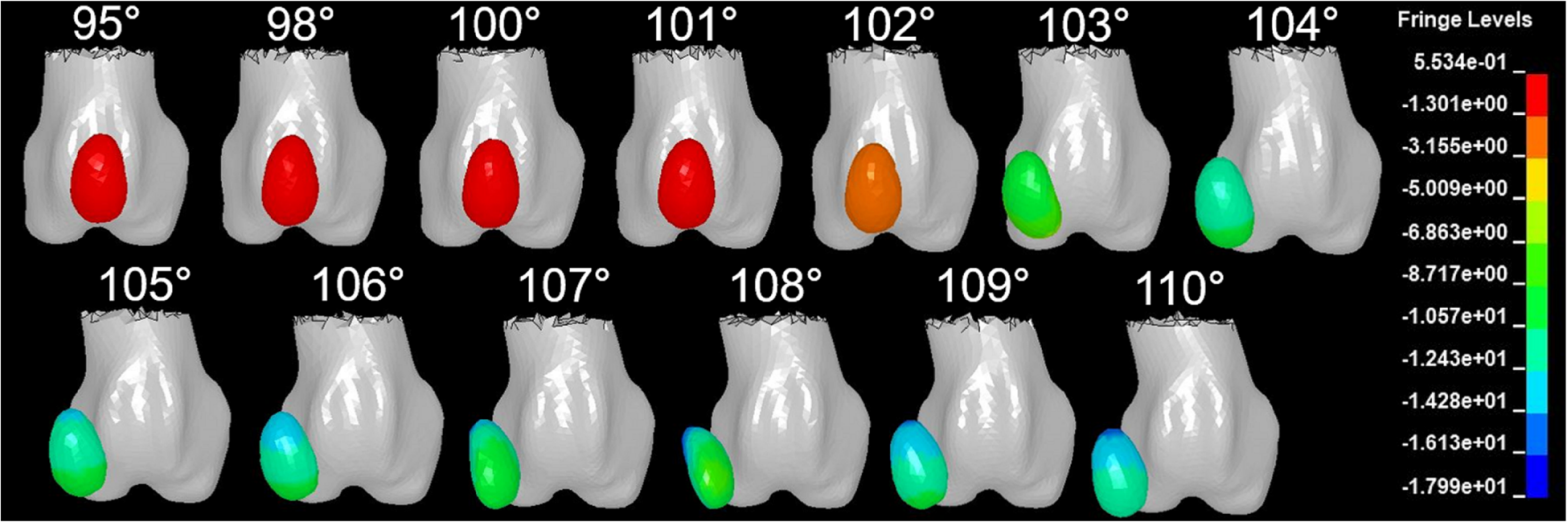
The patellar position in the FE models at the last moment of constant loading on the rectus femoris muscle. The patella tended to luxate with aLDFAs equal to and greater than 103° when other factors were controlled. The fringe levels indicate the magnitude of displacement of the patella from the groove, and the units of measurement are millimeters

**Fig. 2:**
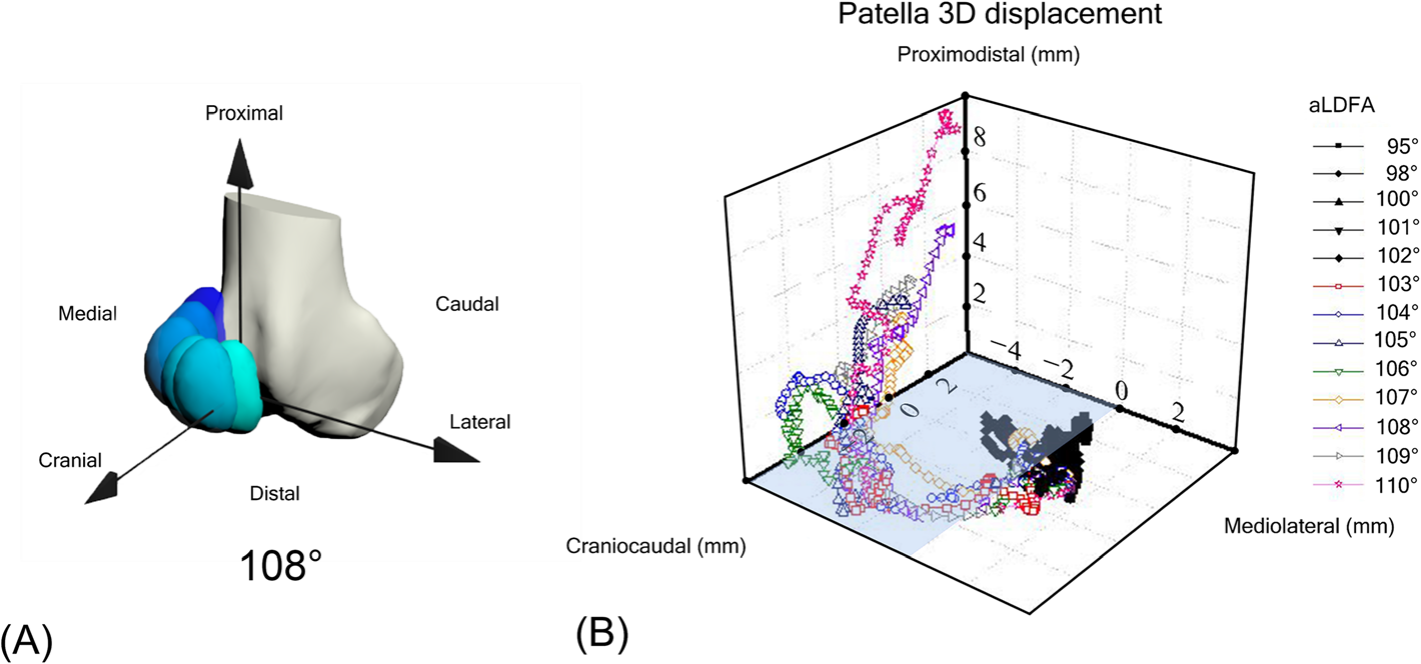
The 3D patellar displacement with a millimeter scale. The x, y, and z-axes represent the mediolateral, craniocaudal, and proximodistal directions, respectively. The black marks grouped together represent the route of relatively stable patella (at aLDFA values ranging from 95° to 102°), while the other colored marks scattered in the craniocaudal and medial directions represent patella at aLDFA values ranging from 103° to 110°. The medial direction of the 3D coordinate graph was represented by the blue surface

In a simulation with the quadriceps constantly activated while stifle angle was fixed at stance phase, beginning with an aLDFA of 103°, the patella began to move medially and luxate (Fig. 1). In three-dimensional coordinate graphs, as aLDFA increased from 103°, the patella was not only displaced medially but also caudally and proximally (Fig. 2). When the aLDFA was equal to or less than 102°, the patella was located in the femoral trochlear groove during the simulation.

### Patellar dynamics according to aLDFA variation

**Fig. 3:**
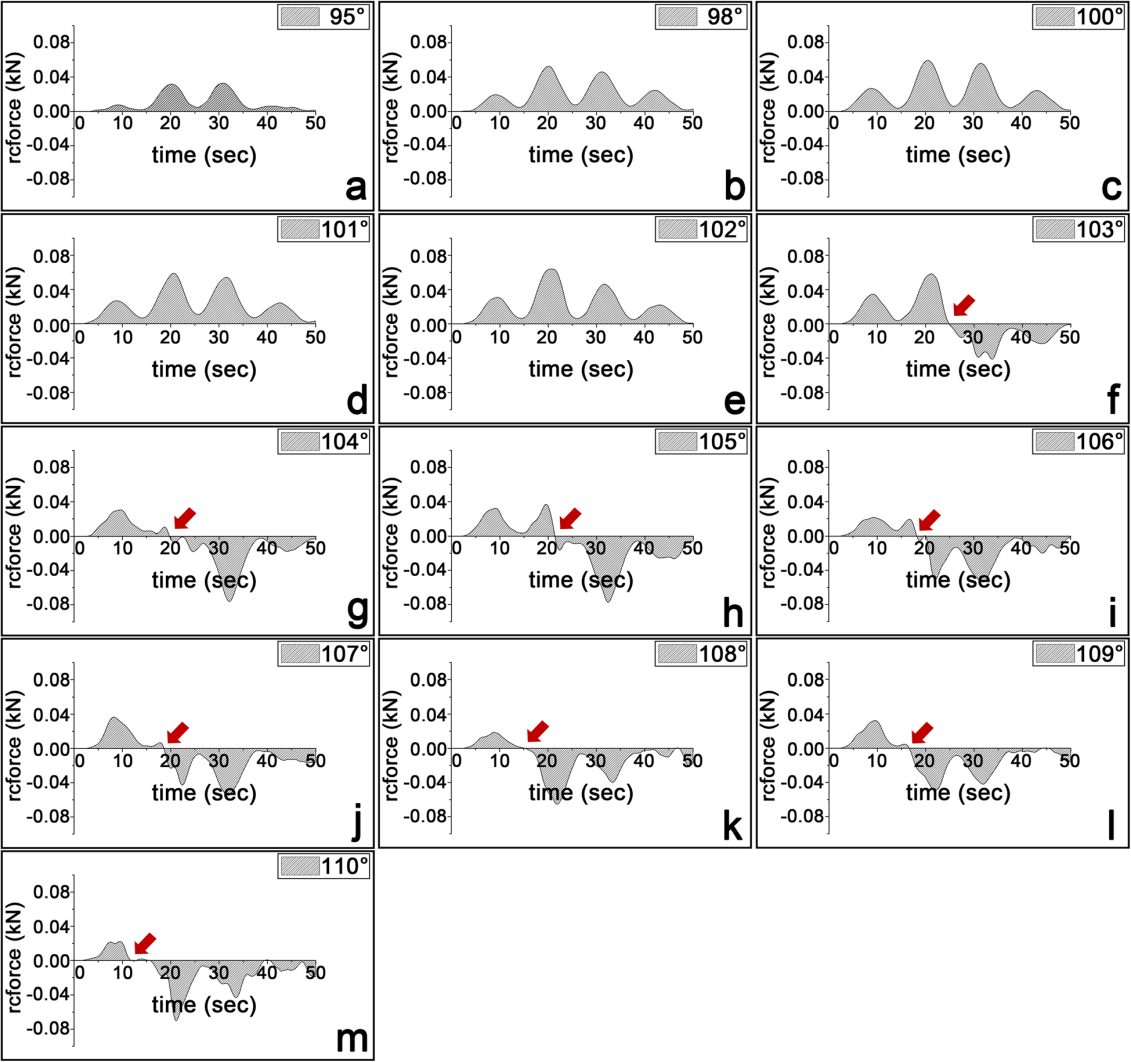
The reaction force (rcforce) of the x-axis between patella and the femoral trochlear groove in the quadriceps-loading simulations. The Rcforce value reaches ‘0’ and decreases to a negative value when the aLDFA is equal to and greater than 103°, respectively, and medial patella luxation occurs when the aLDFA is equal to and greater than 103°. Models corresponding to aLDFAs of 95° (**a**), 98° (**b**), 100° (**c**), 101° (**d**), 102° (**e**), 103° (**f**), 104° (**g**), 105° (**h**), 106° (**i**), 107° (**j**), 108° (**k**), 109° (**l**), and 110° (**m**), and the rcforce units are kilonewtons (1 kN = 1000 N). The red arrow indicates when the rcforce is zero

The reaction force (rcforce) of the x-axis between the patella and the femoral trochlear groove was equal to zero and then decreased to negative values when the aLDFA was equal to and then exceeded 103°, respectively (Fig. 3). The patella began to luxate at 24.90 seconds (sec) with an aLDFA of 103°, 19.80 sec with an aLDFA of 104°, 21.40 sec with an aLDFA of 105°, 20.10 sec with an aLDFA of 106°, 18.60 sec with an aLDFA of 107°, 15.30 sec with an aLDFA of 108°, 16.60 sec with an aLDFA of 109°, and 11.90 sec with an aLDFA of 110°.

## Discussion

The results of this study support the initial hypothesis. In a computer model, severe distal femoral varus with an aLDFA of ≥ 103° caused medial luxation of the patella when other anatomical factors of MPL were controlled. This model found complementary information to anecdotal clinical recommendations of distal femoral osteotomy to correct MPL in large-breed dogs with an aLDFA of ≥ 102° [11, 13]. Interestingly, this is the first report to evaluate the biomechanical effect of distal femoral varus on the stability of the patella.

The biomechanics of the patellofemoral joint are complex, and standardization of the factors related to the joint is difficult in cadaveric and in vivo studies. Moreover, it is considered impossible to experimentally measure the whole three-dimensional state of the forces within the joint. In human medicine, FE-based computer modeling has been broadly used to investigate the biomechanics of knee joints to overcome the limitations and difficulties faced in cadaveric or in vivo studies [15, 16, 19, 22, 23]. Additionally, computer modeling is a method that avoids ethical problems related to animal use and has been increasingly used in veterinary research [20, 21, 24–26]. The current study employed computer modeling to evaluate patellar stability in the femoral trochlea with various degrees of distal femoral varus while controlling factors such as the trochlear groove shape, medial displacement of the tibial tuberosity, and internal rotation of the tibia.

The reaction force (kN) was calculated when the patella contacted the trochlea of the femur. When the aLDFA was larger than 103°, the reaction force became zero and then became a negative value during the simulation (Fig. 3). These aforementioned results indicate that a normal trochlear groove and medial ridge can keep the patella in the appropriate position when the aLDFA is less than or equal to 102°. At aLDFAs exceeding this angle, the trochlea is no longer able to withstand the shear force generated by the quadriceps mechanism between the patella and trochlea. These findings could provide complementary information to clinical studies that reported good functional outcome of 87.5–93% and low recurrence rates of patellar luxation when DFO was applied in high-grade MPL patients with aLDFA of > 100° − 102° [9, 11–14].

A reaction force of 0 kN indicates patella luxation because the two elements, the femur and the patella, no longer react with each other. The negative rcforce was generated because the contact between the femur and the patella occurred in opposite directions. After luxation, the patella remained in lateral contact with the outside of the trochlear groove, and therefore, the rcforce was generated. However, since the patella was already luxated, this phenomenon was not clinically significant.

The total simulation time was set to 50 seconds because whether or not luxation occurred, time was required for the patellar motion to stabilize; thus, the time was set to 50 seconds to allow time for the motion of the patella to stabilize on all of the aLDFA models.

In terms of the outcome of luxation time, the trend was that the greater the aLDFA angle was, the shorter the time until luxation occurred, but some angles did not follow the trend consistently (Fig. 3). Patella luxation occurred at 19 seconds at 104° and approximately 15 seconds at 108°, whereas luxation occurred at approximately 20 seconds at 105° and 106° and 16 seconds at 109°. At an aLDFA of 110°, temporary luxation occurred at 11 seconds, showing an unstable state, and complete luxation occurred at 15 seconds. In the reconstructed canine stifle model used in this study, there was no articular cartilage, synovial fluid, or other soft tissue to buffer the impact generated when the hard cortical bones of the patella and the trochlear groove collided with each other. Therefore, the above time discrepancies may have been due to the rebound phenomenon occurring as soon as the patella hit the medial ridge of the groove. Nevertheless, the fact that soft tissues were not modeled alone does not fully explain why the models with aLDFAs of 104° and 108° dislocated faster than those with other aLDFAs, and we could not determine the exact cause for this particular angle. In addition, patellar rotation, which is not possible with normal anatomy, is present after luxation, and the presence of muscles, ligaments, and the joint capsule prevent this rotation in vivo.

According to the normal aLDFA reported in large-breed dogs [27] and the aLDFA of MPL with a grade of less than 2 reported in small-breed dogs [10, 28], angles of 95° and 98° were considered angles to be unaffected by varus deformities. Therefore, we assumed the critical angle that causes and increases the risk of patellar luxation to be ≥ 100°. However, we did not evaluate an aLDFA of 99°, which was within the range of values reported in grade 3 MPL cases in small-breeds, which was 100.53° ± 2.05°.^10^ Although we can deduce the trend in patellar luxation as the varus angle changes, this point is a limitation of our study and needs to be carefully interpreted.

There are notable limitations of the present study. The FE model was not validated due to the lack of a comprehensive ex vivo testing model or motion analysis method to replicate the entire 3D biomechanics of the canine patellofemoral joint. Even though the quadriceps muscle was loaded during the experiment, the stifle angle was not extended further and remained static as the stance phase. Additionally, since the FE model was derived from an individual Beagle, differences in the stance posture and femoral joint angels according to breed conformation were not considered [27–29]. Moreover, the FE model did not include other factors affecting the MPL, such as cartilage, soft tissue stabilizers such as the components of the lateral aspect (vastus lateralis and lateral patellofemoral ligament), contracture of quadriceps muscle, femoral torsion, shapes of the femoral trochlea, and different positions of the tibial tuberosity. Thus, the results may not be generalizable to all dogs, and the calculated luxation risk may have been overestimated. Therefore, the aLDFA value in MPL obtained here should be considered as an indication of a trend caused by bony deformities and not as an absolute criterion. However, it is interesting that the results of this study are similar with anecdotal clinical recommendations for DFO in large-breed dogs with MPL [9, 13, 14, 30].

## Conclusions

Overall, the results in the present simplified computer model show that distal femoral varus with an aLDFA equal to or greater than 103° leads to MPL. The results based on an FE model are complementary to anecdotal cutoffs for DFO, although it should be applied to clinical patients with caution. The FE model of the canine stifle reported herein requires improvements to further analyze the effects of geometric features such as the breed size, bony shapes and soft tissues.

## Methods

### Asymptomatic geometry preparation

**Fig. 4:**
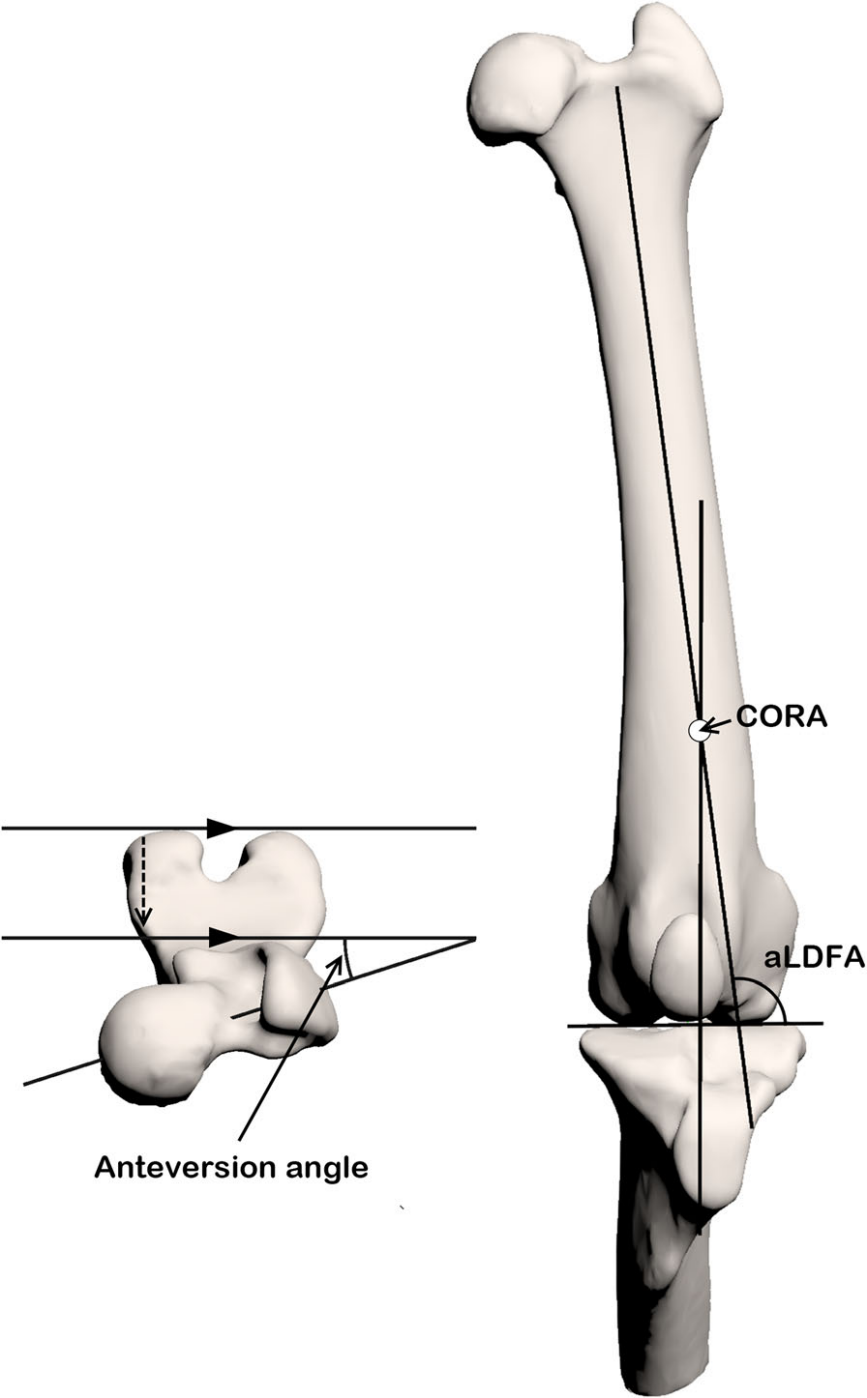
Three-dimensional (3D) reconstructed model of the dog. The anteversion angle of the femur was 16°, and the aLDFA was 95°. The location of the CORA is indicated

This study was approved by the National University of Chungnam’s Institutional Animal Care and Use Committee (Number: CNU-01166). The CT images from a purpose-bred, adult, healthy, male laboratory Beagle dog (provided by the Animal Study Center of Chungnam National University) weighing 10.7 kg were used to create a femoral varus model and were selected as the baseline for the present study. The Beagle dog was released after all the experiment was conducted. No abnormalities were found via physical or orthopedic examinations or radiography in the Beagle dog. A computed tomographic (AlexionTM, Toshiba Medical Systems, Tochigi, Japan) scan of the Beagle dog was performed in the normal-standing position with the hip joint positioned according to that in the procedure previously reported [31]. The CT images were obtained using bone and soft tissue filters at 120 kV and 120 mA, respectively, and a slice thickness of 1 mm. The aLDFA and anteversion angle (AA) of each femur of the Beagle dog were measured (both left and right aLDFAs were 95° with an AA of 16°) (Fig. 4). The three-dimensional (3D) geometries of the left femur, patella, and tibia were extracted and segmented using 3D modeling software (Mimics innovation suite 20, Materialise NV, Leuven, Belgium). Fluoroscopic (BV Pulsera, Philips, Netherlands) images of the Beagle dog were taken while walking on the treadmill at 53 kVp and 3.86 mAs with a pulse rate of 30 frames/s and a pulse width of 11.1 ms. The beam center of the fluoroscope was focused on the stifle mediolaterally. The geometries of these bones were manually aligned to the fluoroscopic images in the stance phase of gait cycle using previously described 3D-to-2D image registration software (JointTrack, University of Florida, http://sourceforge.net/projects/jointtrack/) [32].

### Femoral varus model creation

The asymptomatic normal stifle geometry of the Beagle and the center of rotation of angulation (CORA) were used to create the femoral varus geometry (Fig. 4). The position of CORA, located at the distal one-third of the femur, was based on the computed tomographic (CT) images of a client-owned Maltese dog’s femur, which had an aLDFA of 110°. It was consistent with a previous report of CORA in MPL [8, 9].

The normal femur geometry was then bent at the CORA using meshing software (3dsMax, Autodesk, San Rafael, CA, USA) to create deformed geometries. First, the geometry was scaled to fit the height of the client-owned Maltese dog’s femur and bent at the same CORA level as that of the affected femur until it reached an aLDFA of 110° [33]. After confirming that there was no translocation in the CORA position in the bent model, the remaining angle models were reproduced using a ratio of aLDFA of 95° to 110°.

A total of thirteen different aLDFAs were simulated; normal aLDFA values from 95° (baseline) to 98° and affected angles from 100° to 110° were evaluated in one-degree increments.

**Fig. 5:**
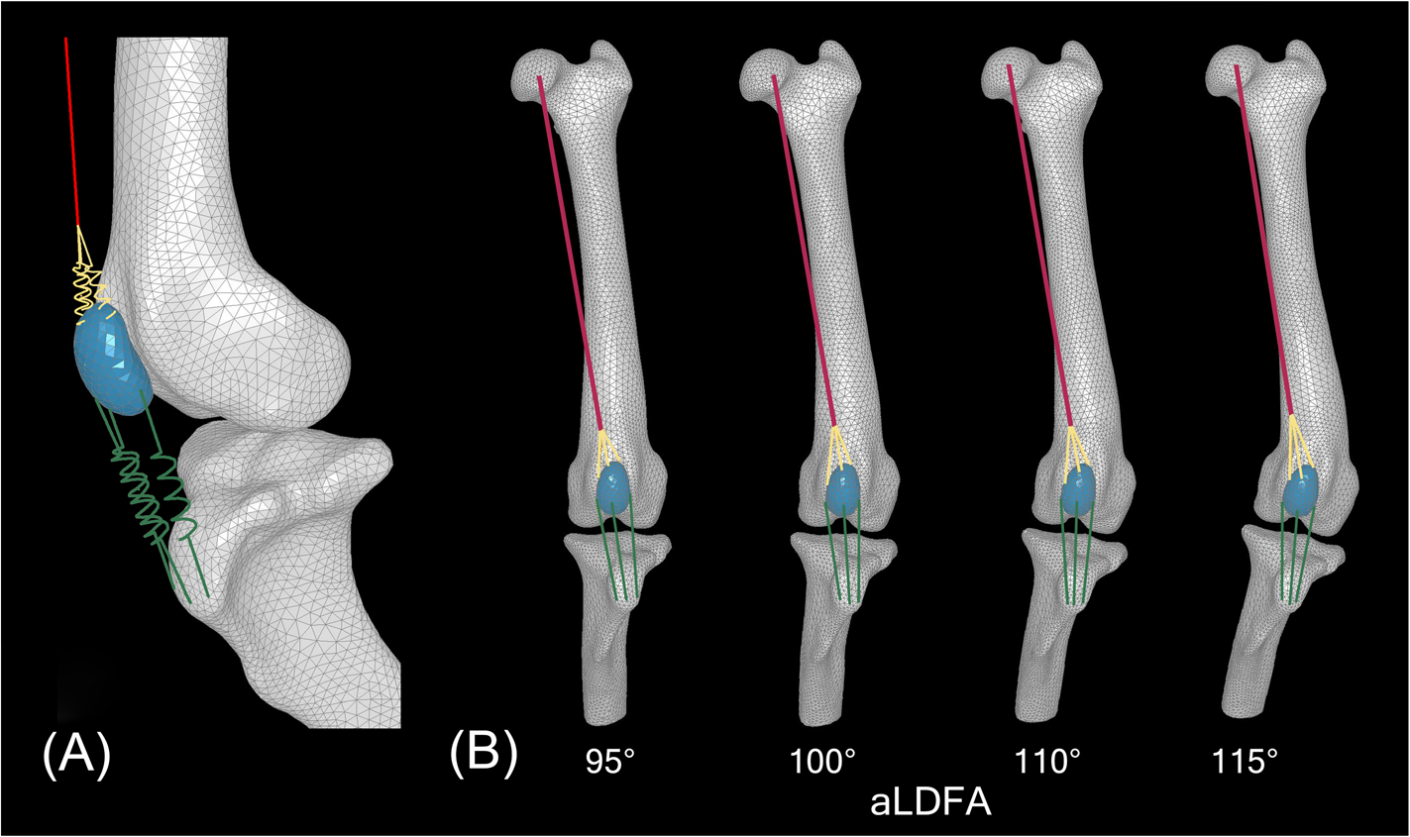
**a**, A geometrically accurate FE model of the asymptomatic canine stifle, with an aLDFA of 95°. The FE model of the canine PF joint includes the rectus femoris muscle (red line), three functional elements of the quadriceps tendon (yellow springs) and the patellar ligament (green springs). The origin of the rectus femoris muscle in the model was set and fixed for all six degrees of motion at a virtual point matching the actual anatomical location of the ilium. **b**, The geometries of four canine stifle models. The most distal part of the femur, including the patella and the trochlear groove, remained anatomically undeformed so that every varus deformation maintained the same geometry of the patella and the trochlear groove

The vertical position of the patellar segment was aligned to the stance phase of the stifle during the gait cycle based on the fluoroscopic images using the 3D-to-2D image registration technique. The angle between the normal femur and tibia segments in the sagittal plane was 127° and remained static during the experiment. The ratio of the length of the patellar ligament to the length of the patella (L:P) was 1.55, which was within the range of values reported in clinically normal large-breed dogs [34]. During this process, the patella and the femoral trochlea remained anatomically undeformed so that every varus deformation had the same patella and trochlear groove geometries (Fig. 5).

### FE model development

Thirteen stifle meshes were created using meshing software. To maintain an equivalent joint plane between the femur and tibia in all models, the tibia and distal epiphysis of the femur were fixed in the model before the femur was bent to create the deformity. Each stifle mesh model had specific tetrahedral solids because the solids were reconstructed by using a 3D stereolithography model before the mesh models were created (Fig. 5b). The mechanical behavior of the patellofemoral joint models was analyzed using Explicit FEM Multi Flexible Body dynamic analysis (LS-DYNA, Livermore Software Technology Corporation, Livermore, CA, USA). The cortical bone was modeled as a solid elastic material with a Young’s modulus of 15.0 GPa and a Poisson’s ratio of 0.3, which were chosen on the basis of the reported values [20, 24]. Cancellous bone was excluded due to its insignificant mechanical role in contact stress predictions, and bones were treated as complete solids in the model [35, 36].

**Fig. 6:**
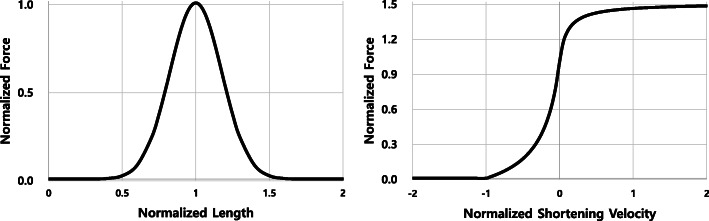
Force-length and force-shortening velocity curves for rectus femoris muscle. The force-length and force-shortening velocity curves were used to calculate the coefficients of the muscle activation force using the initial and current relative length of the muscle, and the shortening velocity of the muscle, respectively

The extensor or quadriceps mechanism includes the quadriceps (rectus femoris, vastus lateralis, vastus medialis, and vastus intermedius muscle), quadriceps tendon, patella, patellar ligament, and tibial tuberosity [2]. In the present study, only the rectus femoris muscle was modeled with a beam element with Hill’s muscle model-based material (Material 156-MUSCLE), provided by LS-DYNA, to represent the quadriceps [37, 38]. The Hill-based model was designed according to Hill’s equation describing the mechanical response of skeletal muscle [39]. In brief, Hill muscle models consist of a contractile element (CE) and parallel element (PE). The contractile element represents the force created by the activation of a muscle, while the parallel element represents the energy stored due to muscle elasticity. The total force F_M_ is defined as the sum of the forces in the contractile element F_CE_ and parallel element F_PE_. The force generated by muscle activations (F_CE_) was calculated as follows:

$$ {\mathrm{F}}_{\mathrm{CE}}=a(t)\times {F}_{max}\times {f}_{FL}\times {f}_{FV} $$

where a(t) is the activation level of the muscle, F_*max*_ is the peak isometric force, and f_FL_ and f_FV_ are normalized force-length and force-shortening velocity functions, respectively. In LS-DYNA, F_*max*_ was calculated automatically using the peak isometric stress, physiological cross-sectional area (PCSA) and f_FL_ and f_FV_ curves (Fig. 6). Thus, F_CE_ can be simply calculated using the current length, shortening velocity, and activation level of the muscle.

The force generated by muscle elasticity (F_PE_) was calculated using the current length of muscle and an exponential relationship as follows:

$$ {\mathrm{F}}_{\mathrm{PE}}=\left\{\begin{array}{c}\epsilon \le 0:\kern3em 0\kern3.5em \\ {}\epsilon >0:\kern1.75em \frac{1}{e^k-1}\left({e}^{\frac{k}{L_{max}}\left(L-1\right)}-1\right)\end{array}\right. $$

where ϵ is the strain of the muscle, L is the current muscle length, L_*max*_ is the maximum strain rate of muscle, and k is a dimensionless shape parameter controlling the rate of rise of the exponential function.

The origin of the rectus femoris muscle model was located based on the CT scan images with a sagittal plane hip angle of 125.2° and constrained for all six degrees of freedom at a virtual point matching the actual anatomical location of the ilium (Fig. 5b).

To transfer more physiological load to the patella, the quadriceps tendon and ligament were separated into three beam elements and configured as linear spring materials with an elastic stiffness of 0.19 kN/mm [22, 40, 41]. The properties of the materials used for the muscle were adapted from previous reports (Table 1) [42, 43].

**Table 1:** The properties of the parameters used to calculate the force generated by the rectus femoris

Parameter	Density	Initial relative length*	Maximum strain rate*	Peak isometric stress	Parallel constant k*	PCSA
Value	1e-5 kg/mm^3^	1.0	2.0	5e-4 GPa	6.0	1850 mm^2^

*Note that parameters are normalized. For example, the peak isometric stress is normalized by dividing the peak isometric force by PCSA (physiological cross-sectional area)

### Boundary conditions

Both the femur and tibia bone models were fully constrained to prevent unnecessary bony rotation or displacement during quadriceps loading, while the patella was unconstrained with three translational and three rotational degrees of freedom. The interaction between the patella and the femoral trochlea groove was defined as a surface-to-surface contact with a friction coefficient of 0.02 [18, 44].

Because a sudden increase in muscle-developed force can cause a large contact force between the patella and femur, which disarticulates the patella unnaturally, thirteen stifle models were simulated with a constant muscle activation level that linearly increased for 0 to 5 sec from 0–20% and remained at 20% from time points 5 to 50 sec, without any changes in the stifle angle.

### Data Analysis

Patellar positions and the reaction force between the patella and the femoral trochlear groove in all FE models were calculated under constant activation of the rectus femoris muscle. When muscle activation was initiated and the first contact point of the patella and the groove was zero, the patellar displacement was expressed as a negative value for the medial direction and a positive value for the lateral direction, according to the global coordinate value.

Then, the critical aLDFA causing medial luxation of the patella was determined. The reaction force (rcforce) (kN) between the patella and the medial trochlear ridge was calculated for each aLDFA during the simulation. The total simulation time was set to 50 seconds to stabilize the motion of the patella in all of the aLDFA models.

## Data Availability

The datasets used and/or analyzed during the current study are available from the corresponding author upon reasonable request.

## Abbreviations

MPL: Medial patella luxation
aLDFAs: Anatomic lateral distal femoral angles
FE: Finite element
DFO: Distal femoral osteotomy
FVA: Femoral varus angle
FEM: Finite element method
Rcforce: Reaction force
IACUC: Institutional Animal Care and Use Committee
CORA: Center of rotation of angulation
CT: Computed tomography
AA: Anteversion angle
CE: Contractile element
SE: Serial element
PCSA: Physiologic cross sectional area
